# Evolving injury profiles amid advancing sport: Musculoskeletal injuries in ringball players

**DOI:** 10.17159/2078-516X/2020/v32i1a8166

**Published:** 2020-01-01

**Authors:** J D Pillay, J Wiggill, B N Mkhwanazi

**Affiliations:** 1 Department of Basic Medical Sciences, Faculty of Health Sciences, Durban University of Technology, South Africa; 2 Department of Chiropractic, Faculty of Health Sciences, Durban University of Technology, South Africa; 3 School of Agricultural, Earth & Environmental Sciences, University of KwaZulu-Natal, Private Bag X101 Scottsville, South Africa

**Keywords:** risk factors, prevalence, mechanism of injury

## Abstract

**Background:**

Ringball, a sport historically derived from conventional basketball and netball, demonstrates the evolution of traditional sport. The variations between these sports may predispose players to different risk factors and consequent injuries and their impact, yet they are usually considered comparably.

**Objective:**

To determine the prevalence of musculoskeletal injuries and to profile injuries based on location, severity, and mechanisms of occurrence. A further objective was to compare the injuries sustained in ringball with that of basketball and netball.

**Methods:**

A questionnaire-based study, administered to 110 ringball players in KwaZulu-Natal, South Africa, was applied. Data were described and compared using frequencies/percentages for categorical variables.

**Results:**

Seventy-six ringball players completed the questionnaire (69% response rate). The prevalence of at least one injury during/after the last or current season was 80% (n=61). The most commonly reported injury was to the foot/ankle (36%; n=33) followed by the knee (29%; n=27) and wrist (9%; n=8). The most common mechanisms of injury reported were landing (15%; n=9), jumping (9%; n=5), goal shooting (7%; n=4), defending (7%; n=4) and collision (7%; n=4).

**Discussion:**

Ringball shares similar injuries to that of basketball/netball, with foot/ankle injuries being the most prevalent. The prevalence of other injuries in ringball differed from basketball/netball, suggesting variations between these sports as a contributor to the injuries described.

**Conclusion:**

The evolution of sport and the modifications in rules/techniques may create a nuanced injury profile to those commonly identified. The combination of a sport’s uniqueness/modification and its similarities to other sports warrants the need for more tailored approaches to injury prevention and a platform for future research.

Ringball, formerly known as ‘korfball’, is a non-contact, family-orientated team sport played by both males and females, comprised of elements from netball and basketball. In 1902, a Dutch primary school teacher developed the sport,^[1]^ then called korfball. The reason for the creation of korfball was to encourage both males and females to participate in the sport on an equal basis.^[1]^ Between 1907 and 1916, korfball was played under the South African Basketball Union and introduced into Afrikaans-speaking schools. Korfball was then made a provincial sport that became nationally and internationally recognised. There are currently approximately 2 500 players in South Africa from all nine provinces who compete against each other annually.^[2]^ In 2007, the name was changed to ‘ringball’ and in 2010 the International Ringball Federation was formed, which introduced ringball to the world.^[2]^

Ringball consists of passing the ball between players with the intention of scoring a goal by shooting it into a basket above the ground, each one of which is situated on either side of the court, as in basketball and netball.^[3]^ These sports have different game rules and court types.^[4]^ For example, ringball has nine players while netball and basketball have seven and five players respectively. In netball, the player receiving the ball, must come to an immediate stop and stay on the same foot on which he/she landed and play the ball without moving this foot.^[5]^ The gameplay of basketball is a continuous flow of running and walking while dribbling the ball in motion.^[6]^ In ringball, once the ball is received, the player is allowed to take an extra two to three steps before stopping, reducing the sudden force on the knee, foot and ankle.^[7]^ These differences can be important in the type and extent of injuries sustained as a result of changes in the flow of motion. Another difference relates to shooting for a goal. In ringball, when a shooter wants to shoot for a goal he/she must be positioned outside the goal area or half circle. The elbow must be slightly flexed below the shoulder and the forearm and hand facing laterally upwards towards the head whilst holding the ball with both hands. The shooter throws the ball with both hands in an underhand motion (from below upwards) towards the head so as to allow for the rotation of the ball towards the goal’s ring. The ball must leave the goal shooter’s hand below the shoulder and must enter the goal net from above.^[7]^ In both basketball and netball, when standing/jumping to throw the ball to the goal net, the shooting elbow is in full flexion with the forearm pronated towards the net. The ball then leaves the hand which is in full flexion.^[5, 6]^ In basketball, any player can score points by throwing the ball through the hoop whether they are inside or outside of the half circle.^[6]^ The further away from the hoop the player is when he/she releases the ball, the greater the number of points that can be scored.^[6]^

Differences in basketball and netball often present with contrasting associated injuries that commonly occur.^[8]^ There is a great similarity in the injuries between the sports, but there are also differences between the most common and least common injuries.^[8]^ The most common injuries reported in basketball are foot/ankle and knee injuries, which make up 40% and 15% of all injuries, respectively.^[9]^ The most common injuries reported in netball are ankle injuries, which make up 38% of injuries and knee injuries making up 29% of all injuries.^[10]^ The least common injuries found in basketball are face/head/neck (14%), hand/arm (10%) and the upper leg/thigh and hip (8%).^[9]^ The least common injuries found in netball are the leg (7%), hand and wrist (7%), shoulder (6%), back (5%), thigh (3%), neck, head, chest (3%) and elbow/arm (3%).^[10]^ Whilst there is similarity in the two sports, differences appear between them in terms of the most common and the least common injuries. As expected, ringball may also show differences, and despite being played for approximately 100 years, there is limited information on the injuries sustained in this sport.^[2]^ The aim of this study was therefore to determine the prevalence of musculoskeletal injuries in ringball players and to profile the different types of musculoskeletal injuries based on location and severity, as well as the mechanisms of injury in the sport. The research undertaken further compares injuries in ringball to those found in basketball and netball given that ringball is derived from both sports. By documenting the risk factors and injuries sustained in terms of location, severity and mechanisms/aetiology of injury, primary measures can be applied to reduce injury occurrence and help manage injuries. Such measures may support the development of guidelines and protocols for sport-specific injury prevention and management.

## Methods

### Study design

A questionnaire-based cross-sectional approach was used in this study.

### Population size and participant recruitment

Participants were recruited from six registered ringball clubs in KwaZulu-Natal (KZN). There were approximately 152 ringball players from 16 teams, all over the age of 16 years. If the participants were under the age of 18 years, the parents or legal guardians completed the parental informed consent, accompanied by an informed permission for minors. The sample size that was required for adequate statistical power was determined to be 110, of which a 70% response rate would obtain appropriate generalisability. Ethical approval was granted by the Institutional Research Ethics Committee (IREC 35/18) at the Durban University of Technology. Gatekeeper permission was obtained from the president of the KZN Ringball Federation. Informed consent was obtained from all participants.

### Measurement tools

This consisted of a self-administered questionnaire which was adapted and contextualised from a validated questionnaire titled: ‘A profile of soccer injuries in selected league amateur indoor and outdoor soccer players in the greater Durban area’.^[11]^ A focus group reviewed the modified questionnaire that was subsequently piloted, with relevant modifications made before it was administered to the participants.

### Data reduction and analysis

A Microsoft Excel spreadsheet was used to capture the data, and then IBM SPSS version 25 was used to analyse the data. A descriptive analysis was undertaken to highlight demographics, frequencies and percentages in the case of categorical variables for the prevalence of the injury, mechanism of the injury, and the location and severity of the injury.

## Results

Of the 110 questionnaires administered, 76 were completed, from 31 (41%) male and 45 (59%) female respondents. This resulted in a response rate of 69%. The mean age reported in males was 29.9±11.3 years and in females was 31.9±12.6 years.

### The prevalence of musculoskeletal injury

**Fig. 1:**
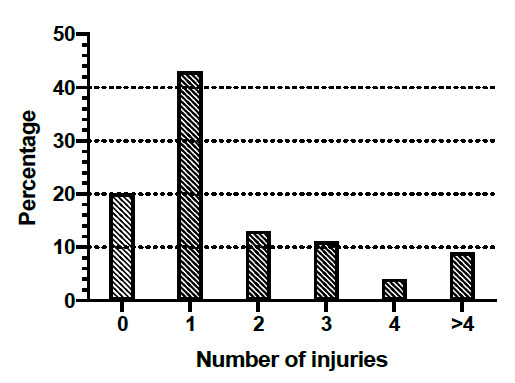
Number of injuries sustained in ringball players (n=61)

All the ringball players who participated in this research study had played at least one season/year of ringball. The prevalence of experiencing an injury over the last and/or current season was 80% (n=61), with some participants reporting more than one injury: 43% reported one injury,13% reported two injuries, 11% reported three injuries, 4% reported four injuries and 9% reported more than four injuries sustained (Fig. 1).

### Location of injury

Of the 93 reported injuries among 61 participants, the most common locations of injuries were the foot/ankle (36%), the knee (29%) and the wrist (9%), respectively. The least common locations of injuries were the head/neck, forearm and genitals at 1% each.

### Mechanisms of injury

Table 1 shows the mechanisms of injury for the first reported injury. The results revealed that the most common mechanisms were incorrect landing 15% (n=9), jumping 9% (n=5), goal shooting 7% (n=4), defending 7% (n=4), collisions 7% (n=4); and other mechanisms 7% (n=4).

### Severity of injury

Severity was estimated by using the number of training sessions or matches missed due to the injury as a proxy. Table 2 shows the extent of the severity of the injuries by considering the number of sessions that were missed. Of the 61 participants who experienced at least one injury, 59 responded to the question on the number of training sessions missed. Hence some participants may have been injured as a result of training sessions missed but did not answer this question.

Fifty-eight players reported on the number of competitive matches missed due to their first injury. Of the 58 players, 10% (n=6) reported missing one competitive match, 16% (n=9) two matches, 12% (n=7) three matches, 5% (n=3) four matches and 24% (n=14) more than four competitive matches.

Another estimate that was used as a proxy to determine the injury severity was the number of days that were missed due to an injury. Table 3 shows the extent of the severity of the injuries by taking into account the number of days that were missed in each case. The number of days that the players were unavailable for training and competitive matches for their reported injury was highlighted.

A total of 56 participants reported the number of days they were unavailable for training. Out of the 56 participants, 23% (n=13) did not miss a training session, 14% (n=8) were unavailable for one to three days, 16% (n=9) were unavailable for four to seven days, 16% (n=9) were unavailable for one to two weeks, 9% (n=5) were unavailable for three to four weeks, and 21% (n=12) were unavailable for more than one month.

There were 52 participants who reported the number of days they were unavailable for competitive matches for their first reported injury. Of the 52 participants, 29% (n=15) did not miss a competitive match, 8% (n=4) were unavailable for one to three days, 15% (n=8) were unavailable for four to seven days, 19% (n=10) were unavailable for one to two weeks, 8% (n=4) were unavailable for three weeks, and 21% (n=11) were unavailable for more than one month.

## Discussion

### The prevalence of injury in ringball compared to basketball and netball

The prevalence of injury in this study with regard to at least one injury over the last/current season was 80% (n=61) (Fig. 1). There were 93 reported injuries amongst 61 injured participants (Table 2) of which the most common locations of injuries were the foot/ankle at 36%, followed by the knee at 29% and wrist 9%, respectively. Studies conducted by Pillay and Frantz and Ferreira and Spamer on netball revealed the prevalence of injuries to the foot/ankle as 38% and 39% respectively.^[10, 12]^ Additionally, the injury prevalence to the knee was 27% and 28%, respectively. These studies revealed similar results to this present study of the most common areas of injuries. Hampton also reported that the foot/ankle (64%) and knee (15%) were mostly injured while reporting prevalence rates that were markedly different.^[13]^ Mckay et al. also reported the ankle (30%) as the most commonly injured area in netball; however, the study reported the hand (21%) as the second most commonly injured area and the knee (18%) as the third most commonly injured area.^[8]^ The studies conducted by Andreoli et al. and Borowski et al. reporting on injuries sustained in basketball were similar regarding the types of most commonly occurring injuries but varied with respect to the prevalence rates.^[8, 9, 14]^. The studies demonstrated foot/ankle injuries at a prevalence of 22% and 40%, respectively, and the prevalence of knee injuries at 18% and 15%, respectively.

A significant association between injury and not warming up before training (p=0.013) and competitive matches (p=0.044) was found. Several studies have demonstrated the benefit of warming up prior to sport participation in reducing injury.^[15–17]^ The limited exposure to warming up in this sample group may have been a contributing factor to the higher injury prevalence observed. This study therefore highlights that coaches and players should pay special attention to warming up before competitive matches and training sessions as a simple and cost-effective strategy that may reduce injury.

### The mechanisms of injuries

The results of this study showed incorrect landing (15%) to be the most common mechanism of injury and jumping (9%) to be the second most common mechanism of injury. Basketball can be a more physical game on the court compared to netball and ringball, which provides a possible explanation as to why some of the results may differ, particularly the regard to defensive rebounding. Pillay and Frantz, Hopper et al., and Mckay et al., all reported similar results to this present study.^[8, 10, 18]^ The studies reported that landing incorrectly was one of the most common mechanisms of injury. In addition, Hopper et al. and Mckay et al. both reported that contact with another player (collision) was also one of the commonly reported mechanisms of injury.^[8, 18]^ The similarities of injuries can be explained by the general gameplay that involves repetitive jumping, landing and sudden sprints in basketball, netball and ringball. In order to implement the most effective preventative measures, investigating the exact cause of each player’s pain/injury is necessary. This may require the use of three-dimensional kinematic data, in addition to pain prevalence data related to injury location as highlighted by Goosey-Tolfrey et al.^[19]^ In obtaining three-dimensional kinematic data, Goosey-Tolfrey et al. revealed that some players generated greater angular velocity of the wrist at the release of the free throw, whilst others generated greater shoulder flexion angular velocity at the release of the free throw.^[19]^ Whilst these findings were related to wheelchair basketball players, the findings concluded that different kinematic strategies may be the basis of the prevalence of pain and injury in sport. It is also important for both coaches and practitioners to identify and address biomechanical errors or deficiencies among athletes to ensure a full recovery or prevent injury.^[19]^ Re-examining game technique and rules may also be relevant.

### Severity of injuries

A total of 9% of participants (n=5) were unavailable for training for three to four weeks, 21% (n=12) were unavailable for more than one month, 8% (n=4) were unavailable for competitive matches for three to four weeks and 21% (n=11) were unavailable for more than one month. Dick et al. reported that 18% of the participants were restricted from activity, i.e. both competitive matches and practices, for more than ten days.^[20]^ This comparison is somewhat different from these authors reporting approach as there is overlap between days and weeks compared between these studies.^[20]^ A further explanation for this difference may be due to the study period and amount of injuries reported.^[20]^ Dick et al. performed their study over a 16-year period, hence providing more longitudinal data, whilst the current study was conducted over a three to four month period reporting cross-sectional data.

Notwithstanding this, these authors’ study indicates a need for possible modification of training regimes or protective support during sport performance. For example, Sitler et al. reported in their study conducted on United States Military Academy cadet basketball players that ankle injuries were remarkably reduced by ankle stabilisers.^[21]^ Baker proposed that bracing the knee to prevent injury provided little knee joint ligament protection, although the ankle, thumb and elbow joints can be stabilised adequately.^[22]^ Barret et al. showed significant differences between ankle sprains and high- versus low-top shoes.^[23]^

### Key findings

- A prevalence of at least one injury in ringball players was 80%. The possibility of having a second injury was 33% and a third injury was 9%.
- The most common locations injured were the foot/ankle (36%), knee (29%) and wrist (7%).
- The main mechanism of injury for the first injury was incorrect landing at 15%, jumping 9%, goal shooting 7%, defending 7%, collision 7% and other mechanisms 7%.

### Strengths of the study

According to these authors’ knowledge, this is the first study on the epidemiology of musculoskeletal injuries in ringball players. The study is also the first in South Africa to obtain prevalence data of musculoskeletal injuries in ringball.

### Limitations and recommendations

The study was limited to one province in South Africa and may not be representative of the entire country. External physical factors, such as the court surfaces, individual factors, such as Body Mass Index, as well as environmental factors, such as the season of the year, should be considered as part of the analyses/associations drawn. A larger population size should be included in future studies of the epidemiology of musculoskeletal injuries of ringball players in KwaZulu-Natal or a study on the ringball players of all provinces in South Africa. Studies should also investigate the court surfaces and consequent incidences of injuries sustained. More attention could be placed on the knowledge that the ringball players have of healthcare professionals and the role they play with regard to injuries, and the most effective treatment protocols for their injuries. Analyses of body composition and other individual measures can be assessed to determine possible associations.

## Conclusion

Constant evolution of sport, through modifications in applied rules and techniques, as well as through the advent of new sports developing from existing ones, can create a more nuanced injury profile to those commonly identified. The discourse between a sport’s uniqueness/modification and its similarities to other sports warrants the need for a more tailored approach to injury prevention and, as such, an important platform for further research. By documenting the risk factors and injuries sustained in terms of location, severity, mechanism of injury etc., can prevent/reduce further injuries from occurring and better managing these injuries through tailored guidelines and protocols for injury prevention and management specific to the sport.

## Figures and Tables

**Table 1:** Mechanisms of injury for first reported injury (n=59)

Mechanism of injury	Count (n)	Percentage (%)
Landing: competitive	9	15
Jumping: competitive	5	9
Collision: competitive	4	7
Defending competitive	4	7
Goal shooting: competitive	4	7
Other: competitive	4	7
Ball throw: competitive	3	5
Running/short sprints: competitive	2	3
Turning: competitive	2	3
Defending: training	2	3
Jumping: training	2	3
Goal shooting: competitive and training	2	3
Landing: competitive and training	2	3
Running/short sprints: competitive and training	2	3
Running and turning: competitive	2	3
Landing and jumping	2	3
Landing: training	1	2
Defending: competitive and training	1	2
Goal shooting: training	1	2
Goal shooting: competitive and training	1	2
Ball throw and goal shooting	1	2
Landing and running/short sprints: competitive and training	1	2
Ball throw training, collision training, defending competitive, goal shooting competitive, jumping training, landing training, overexertion competitive and running/short sprints competitive	1	2
Collision competitive, jumping competitive and running/ short sprints	1	2

**Total**	**59**	**100**

**Table 2:** Number of training sessions and competitive matches missed in the last season as a result of injury (n=59)

		Number of sessions missed					

		0	1	2	3	4	>4
Training sessions missed last season as a result of first injury	n	21	8	9	4	4	13
	%	36	14	15	7	7	22

Training sessions missed last season as a result of second injury	n	11	1	2	2	3	3
	%	50	5	9	9	14	14

Training sessions missed last season as a result of third injury	n	4	1	1	0	0	0
	%	67	17	17	0	0	0

Competitive matches missed last season as a result of first injury	n	19	6	9	7	3	14
	%	33	10	16	12	5	24

Competitive matches missed last season as a result of second injury	n	9	2	3	7	1	2
	%	38	8	13	29	4	8

Competitive matches missed last season as a result of third injury	n	4	0	1	0	0	0
	%	80	0	20	0	0	0

n, number of participants; %, percentage of training or match sessions missed

**Table 3:** Number of days that participants were unavailable during training sessions and competitive matches (n=52)

		Number of days/weeks unavailable					

		None	1–3 days	4–7 days	1–2 weeks	3–4 weeks	>1 month
Number of days unavailable for training due to first injury	n	13	8	9	9	5	12
	%	23	14	16	16	9	21

Number of days unavailable for training due to second injury	n	9	0	2	6	3	2
	%	41	0	9	27	14	9

Number of days unavailable for training due to third injury	n	2	0	0	1	1	1
	%	40	0	0	20	20	20

Number of days unavailable for competitive matches due to first injury	n	15	4	8	10	4	11
	%	29	8	15	19	8	21

Number of days unavailable for competitive matches due to second injury	n	6	1	1	6	2	2
	%	33	6	6	33	11	11

Number of days unavailable for competitive matches due to third injury	n	2	0	0	1	1	1
	%	40	0	0	20	20	20

n, number of participants; %, percentage of training or match days unavailable
